# Supplementary material for: Organization and hierarchy of the human functional brain network lead to a chain-like core
Source: Sci Rep. 2017 Jul 7;7:4888. doi: 10.1038/s41598-017-04716-3 (PMC5501790; doi:10.1038/s41598-017-04716-3)
Supplement: Supplementary file 1 — Supplementary Material [file 41598_2017_4716_MOESM1_ESM.pdf]

# Organization and hierarchy of the human functional brain network lead to a chain-like core - Supporting Information

Rossana Mastrandrea*[1], Andrea Gabrielli[1,2], Fabrizio Piras[3,4], Gianfranco Spalletta[4,5], Guido Caldarelli[1,2]
Tommaso Gili[3,4]

**1 IMT School for Advanced Studies, Lucca, piazza S. Ponziano 6, 55100 Lucca, Italy**
**2 Istituto dei Sistemi Complessi (ISC) - CNR, UoS Sapienza, Dipartimento di Fisica, Universitá "Sapienza"; P.le Aldo Moro 5, 00185 - Rome, Italy**
**3 Enrico Fermi Center, Piazza del Viminale 1, 00184 Rome, Italy**
**4 IRCCS Fondazione Santa Lucia, Via Ardeatina 305, 00179 Rome, Italy**
**5 Menninger Department of Psychiatry and Behavioral Sciences, Baylor College of Medicine, Houston, Tx, USA**
**∗ E-mail: rossana.mastrandrea@imtlucca.it**

## AutoAutomated Anatomical Labeling: numbers and colors

| Numbers | Frontal Lobe ● |
|---|---|
| 1-2 | Precentral gyrus (PRE) |
| 3-4 | Superior frontal gurus, dorsolateral (F1) |
| 5-6 | Superior frontal gyrus, orbital (F1O) |
| 7-8 | Middle frontal gyrus (F2) |
| 11-12 | Inferior frontal gyrus, opercular (F3OP) |
| 13-14 | Inferior frontal gyrus, triangular (F3OP) |
| 15-16 | Inferior frontal gyrus, orbital (F3O) |
| 17-18 | Rolandic operculum (RO) |
| 19-20 | Supplementary motor area (SMA) |
| 21-22 | Olfactory cortex (OC) |
| 23-24 | Superior frontal gyrus, medial (F1M) |
| 25-26 | superior frontal gyrus, media orbital (F1MO) |
| 27 - 28 | Gyrus rectur (GR) |
| 69- 70 | Paracentral lobule (PCL) |
| **Numbers** | **Insula ●** |
| 29-30 | Insula (IN) |
| **Numbers** | **Cingulate ●** |
| 31-32 | Cingulate gyrus, anterior part (ACIN) |
| 33-34 | Cingulate gyrus, mid part (MCIN) |
| 35-36 | Cingulate gyurs, posterior part (PCIN) |
| **Numbers** | **Temporal Lobe ●** |
| 39-40 | Parahippocampus (PHIP) |
| 55-56 | Fusiform gyrus (FUSI) |
| 79-80 | Heschl gyrus (HES) |
| 81-82 | Superior temporal gyrus (T1) |
| 83-84 | Temporal pole: superior (T1P) |
| 85-86 | Middle temporal gyrus (T2) |
| 87-88 | Temporal pole: middle (T2P) |
| 89-90 | Inferior temporal gyrus (T3) |
| **Numbers** | **Occipital Lobe ●** |
| 43-44 | Calcarine fissure and surrounding cortex (V1) |
| 45-46 | Cuneus (Q) |
| 47-48 | Lingual gyrus (LING) |

*Continued on next page*

| Table 1 – *Continued from previous page* | |
|---|---|
| 49-50 | Superior occipital lobe (O1) |
| 51-52 | Middle occipital lobe (O2) |
| 53-54 | Inferior occipital lobe (O3) |
| **Numbers** | **Parietal Lobe** ● |
| 57-58 | Postcentral gyrus (POST) |
| 59-60 | Superior parietal gyrus (P1) |
| 61-62 | Inferior parietal gyrus (P2) |
| 63-64 | Supramarginal gyrus (SMG) |
| 65-66 | Angular gyrus (AG) |
| 67-68 | Precuneus (PQ) |
| **Numbers** | **Deep Grey Matter** ● |
| 37-38 | Hippocampus (HIP) |
| 41-42 | Amygdala (AMYG) |
| 71-72 | Caudate nucleus (CAU) |
| 73-74 | Putamen (PUT) |
| 75-76 | Pallidum (PAL) |
| 77-78 | Thalamus (THA) |
| **Numbers** | **Cerebellum** ● |
| 91-92 | Cerebellum crus 1 |
| 93-94 | Cerebellum crus 2 |
| 95-96 | Cerebellum 3 |
| 97-98 | Cerebellum 4-5 |
| 99-100 | Cerebellum 6 |
| 101-102 | Cerebellum 7 |
| 103-104 | Cerebellum 8 |
| 105-106 | Cerebellum 9 |
| 107-108 | Cerebellum 10 |
| 109 | Vermis 1-2 |
| 110 | Vermis 3 |
| 111 | Vermis 4-5 |
| 112 | Vermis 6 |
| 113 | Vermis 7 |
| 114 | Vermis 8 |
| 115 | Vermis 9 |
| 116 | Vermis 10 |

Tab. 1: **Automated Anatomical Labeling (AAL) atlas.** The first column indicates the label number, the second the name and abbreviation of each Region Of Interest (ROI). For paired structures the first and second numbers refer to the left and right part, respectively. Colors characterize anatomical regions.

## Inter-subject variability

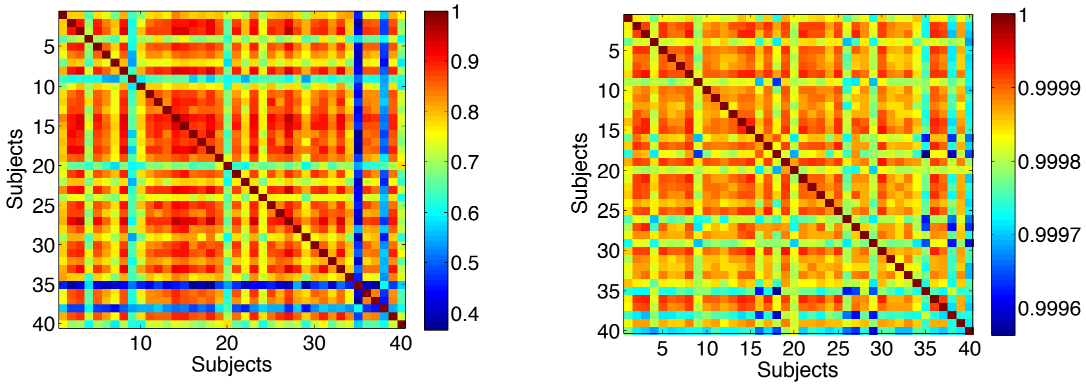


(a) (b)

Fig. S1: **Inter-subjects comparison.** (a) Pairwise cosine similarity between the correlation matrices of the 40 subjects; (b) Pairwise cosine similarity between the average correlation matrices obtained with a Jackknife resampling technique.

## Parcellation: 116 ROIs

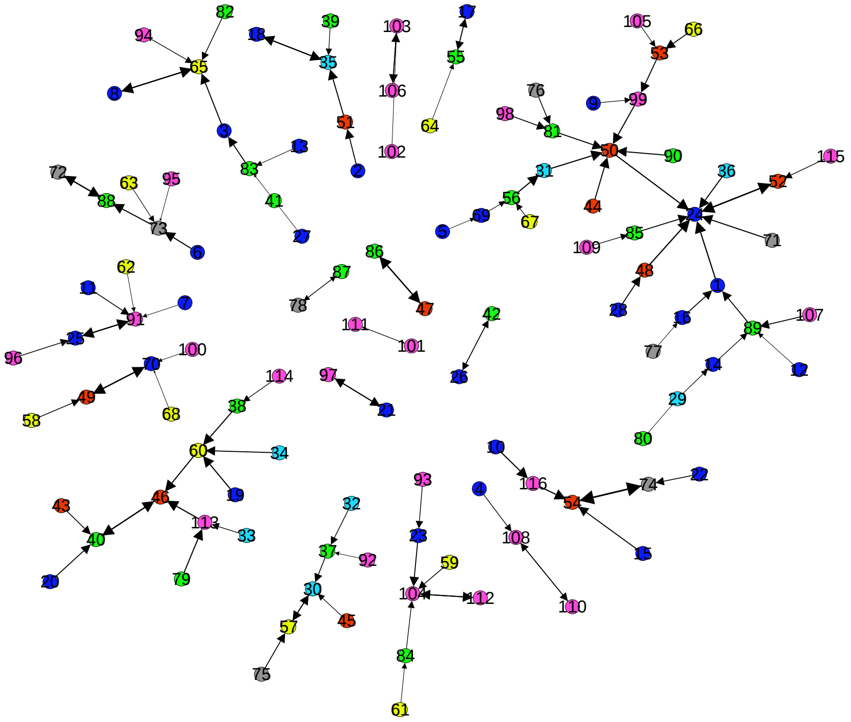

Fig. S2: **Maximum Spanning Forest of a randomization of the real network anatomically parcellized in 116 ROIs.** Arrows indicate that the ROI-source is maximally correlated with the ROI-target. Colors represent anatomical regions according to the grouping of AAL parcellation in 116 ROIs (fig.3(a)) : ● Frontal Lobe; ● Insula; ● Cingulate; ● Temporal Lobe; ● Occipital Lobe; ● Parietal Lobe; ● Deep Grey Matter; ● Cerebellum.

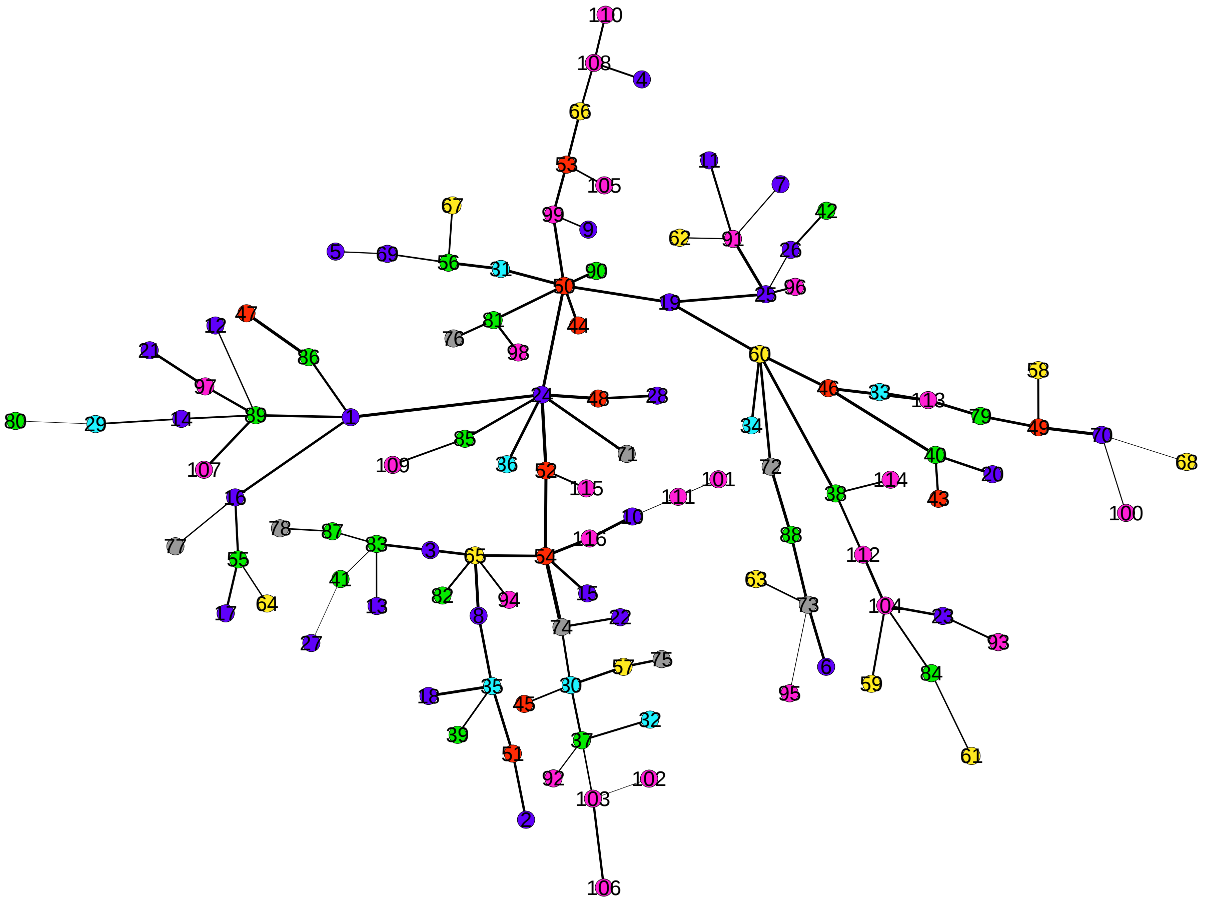

Fig. S3: **Maximum Spanning Tree of a randomization of the real network anatomically parcellized in 116 ROIs.** Colors represent anatomical regions according to the grouping of AAL parcellation n 116 ROIs (fig. 3(a)) :● Frontal Lobe; ● Insula; ● Cingulate; ● Temporal Lobe; ● Occipital Lobe; ● Parietal Lobe; ● Deep Grey Matter; ● Cerebellum.

## Sub-parcellation: 276 ROIs

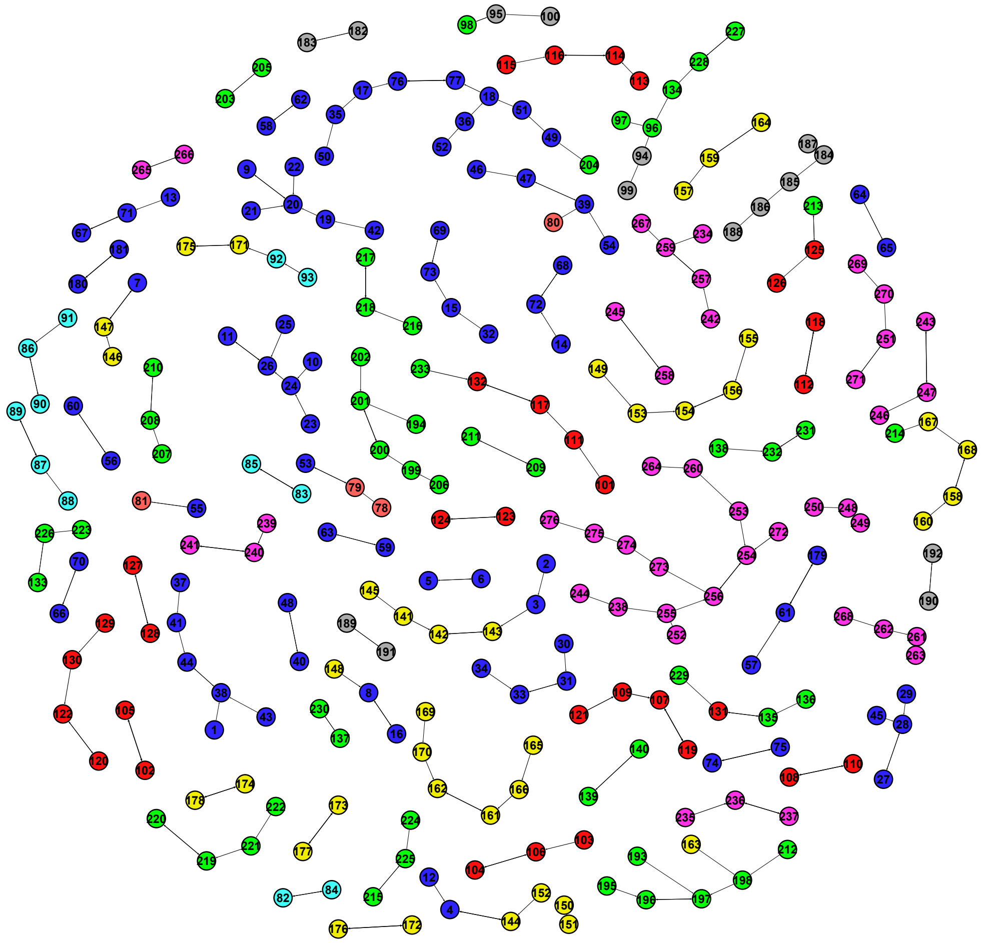

Fig. S4: **Maximum Spanning Forest of the real network anatomically parcellized in 276 ROIs.** Arrows indicate that the ROI-source is maximally correlated with the ROI-target. Colors represent anatomical regions according to the grouping of AAL parcellation in 276 ROIs. The anatomical regions are the same of fig. 3(a), the only difference is in the number of ROIs each region is divided into (see Methods for details): ● Frontal Lobe; ● Insula; ● Cingulate; ● Temporal Lobe; ● Occipital Lobe; ● Parietal Lobe; ● Deep Grey Matter; ● Cerebellum.

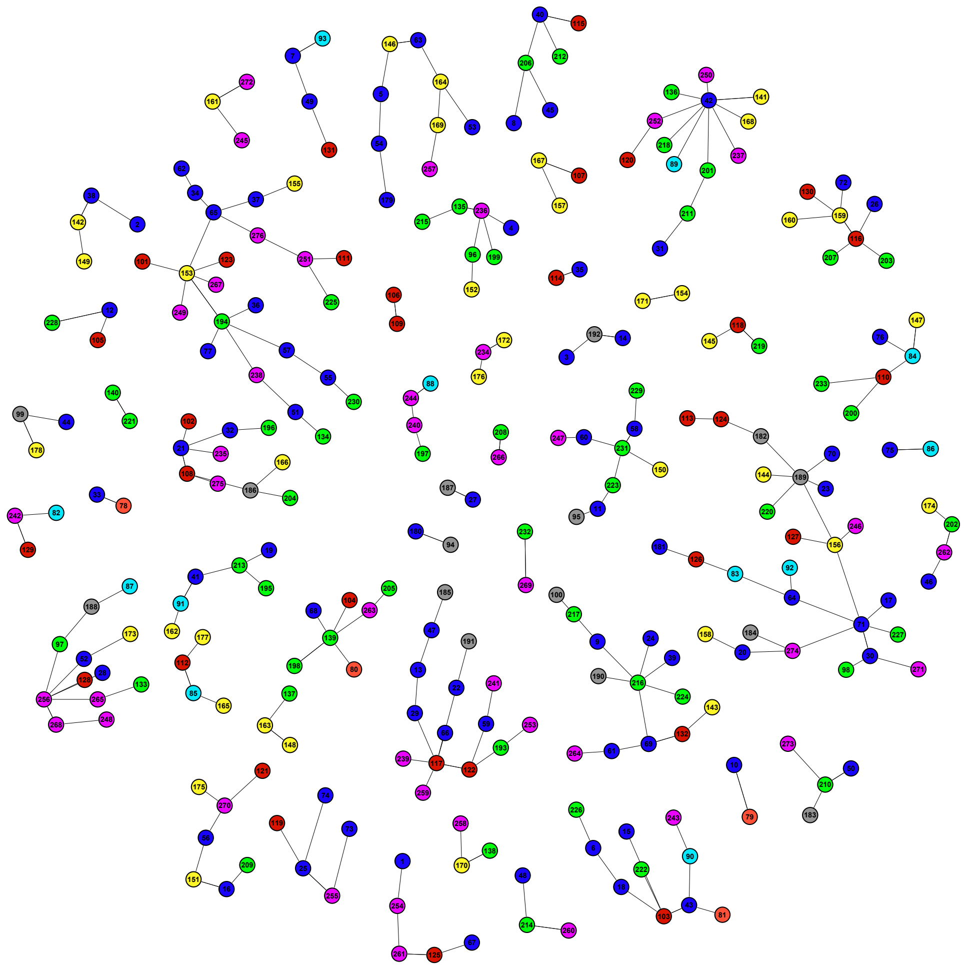

Fig. S5: **Maximum Spanning Forest of a randomization of the real network anatomically parcellized in 276 ROIs.** Arrows indicate that the ROI-source is maximally correlated with the ROI-target. Colors represent anatomical regions according to the grouping of AAL parcellation in 276 ROIs. The anatomical regions are the same of fig. 3(a), the only difference is in the number of ROIs each region is divided into (see Methods for details):● Frontal Lobe; ● Insula; ● Cingulate; ● Temporal Lobe; ● Occipital Lobe; ● Parietal Lobe; ● Deep Grey Matter; ● Cerebellum.

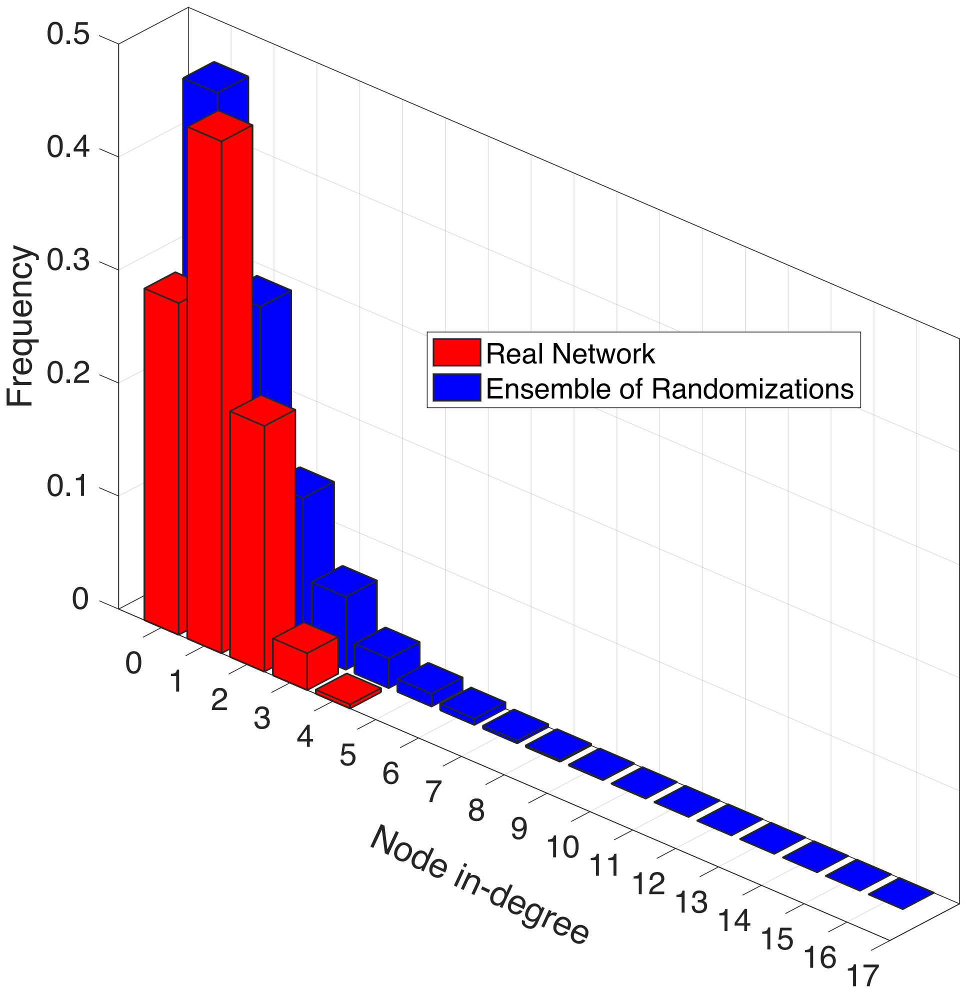


Fig. S6: **Node in-degree distribution of the MSF**. Comparison between the real brain network and the ensemble of its randomizations for the anatomical parcellation of the brain in 276 ROIs.

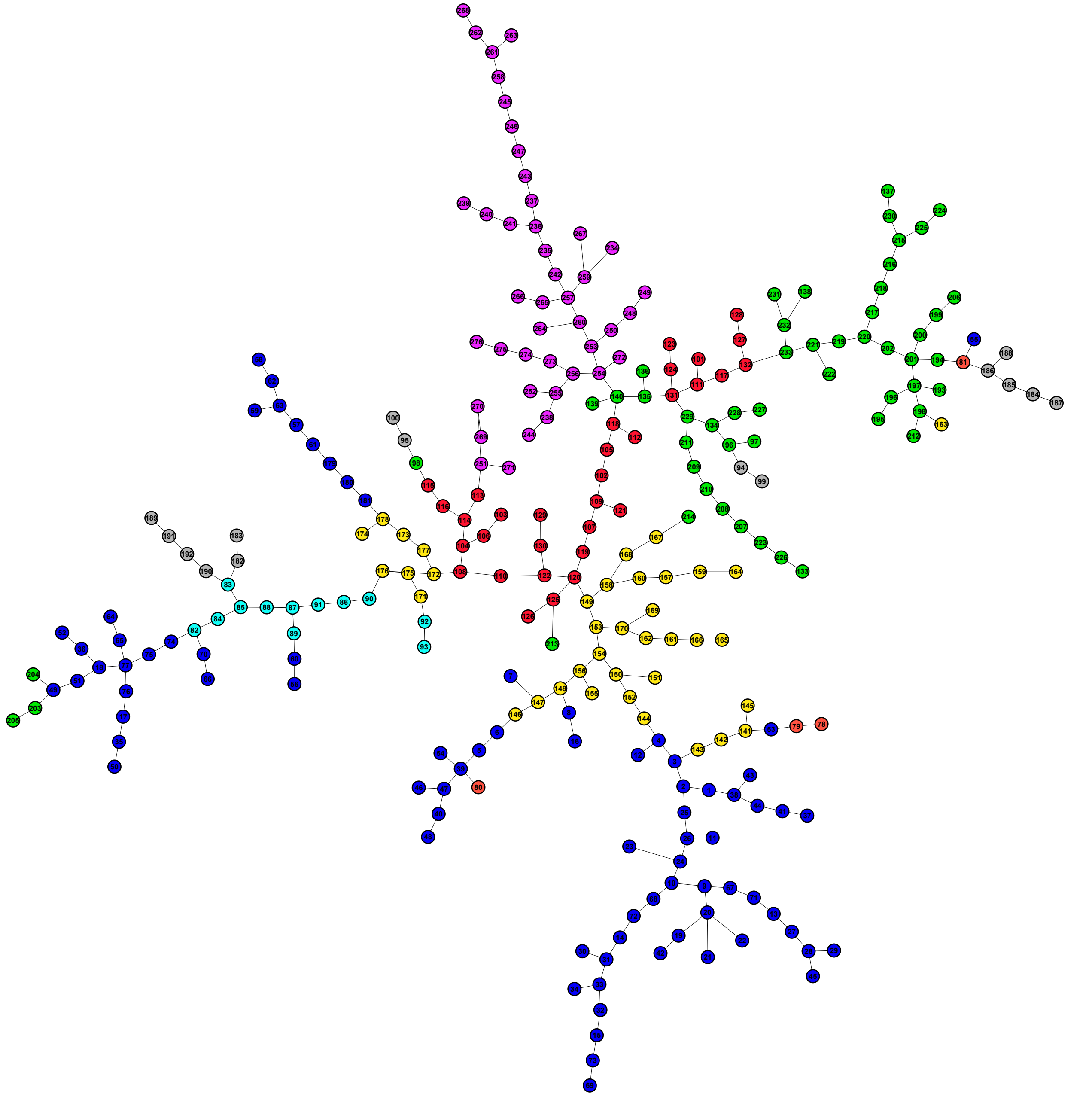

Fig. S7: **Maximum Spanning Tree of the real network anatomically parcellized in 276 ROIs.** Colors represent anatomical regions according to the grouping of AAL parcellation in 276 ROIs. The anatomical regions are the same of fig. 3(a), the only difference is in the number of ROIs each region is divided into (see Methods for details) : ● Frontal Lobe; ● Insula; ● Cingulate; ● Temporal Lobe; ● Occipital Lobe; ● Parietal Lobe; ● Deep Grey Matter; ● Cerebellum.

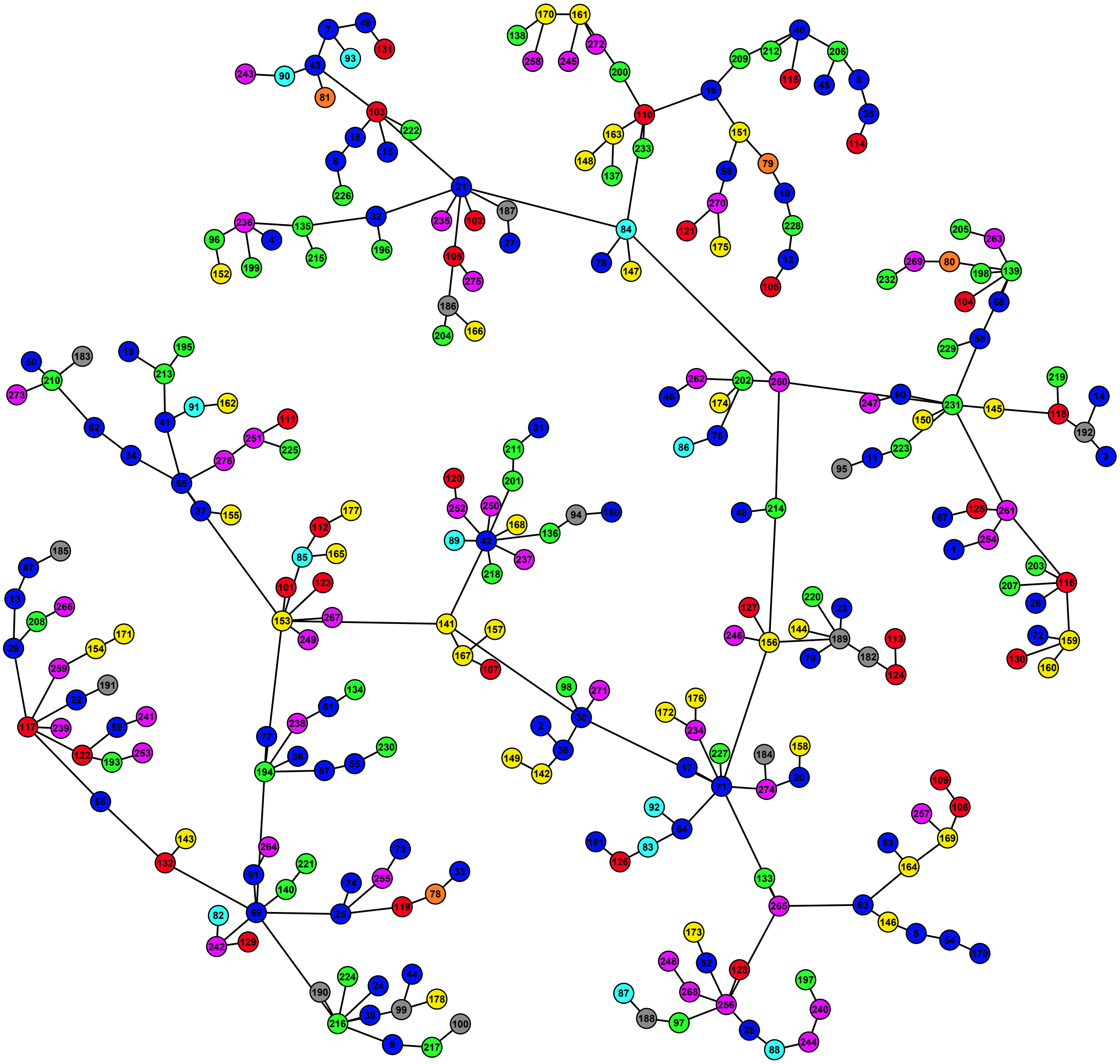

Fig. S8: **Maximum Spanning Tree of a randomization of the real network anatomically parcellized in 276 ROIs.** Colors represent anatomical regions according to the grouping of AAL parcellation in 276 ROIs. The anatomical regions are the same of fig. 3(a), the only difference is in the number of ROIs each region is divided into (see Methods for details) : ● Frontal Lobe; ● Insula; ● Cingulate; ● Temporal Lobe; ● Occipital Lobe; ● Parietal Lobe; ● Deep Grey Matter; ● Cerebellum.

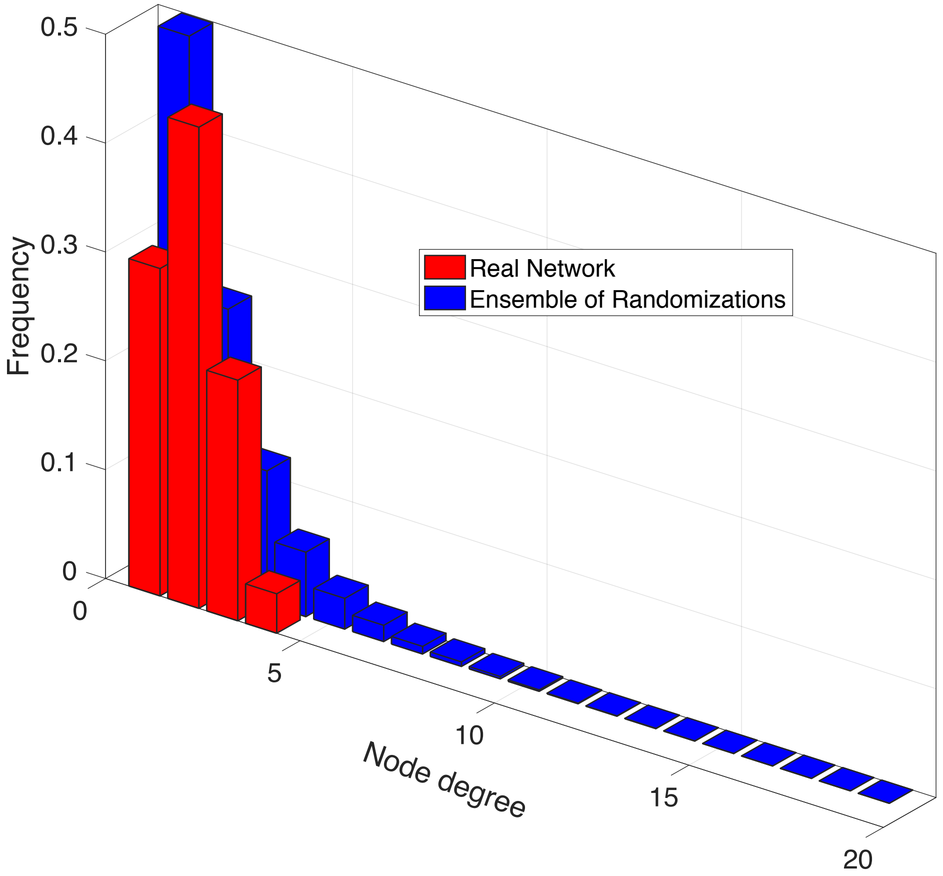


Fig. S9: **Node degree distribution of the MST**. Comparison between the real brain network and the ensemble of its randomizations for the anatomical parcellation of the brain in 276 ROIs.

## Sub-parcellation: 531 ROIs

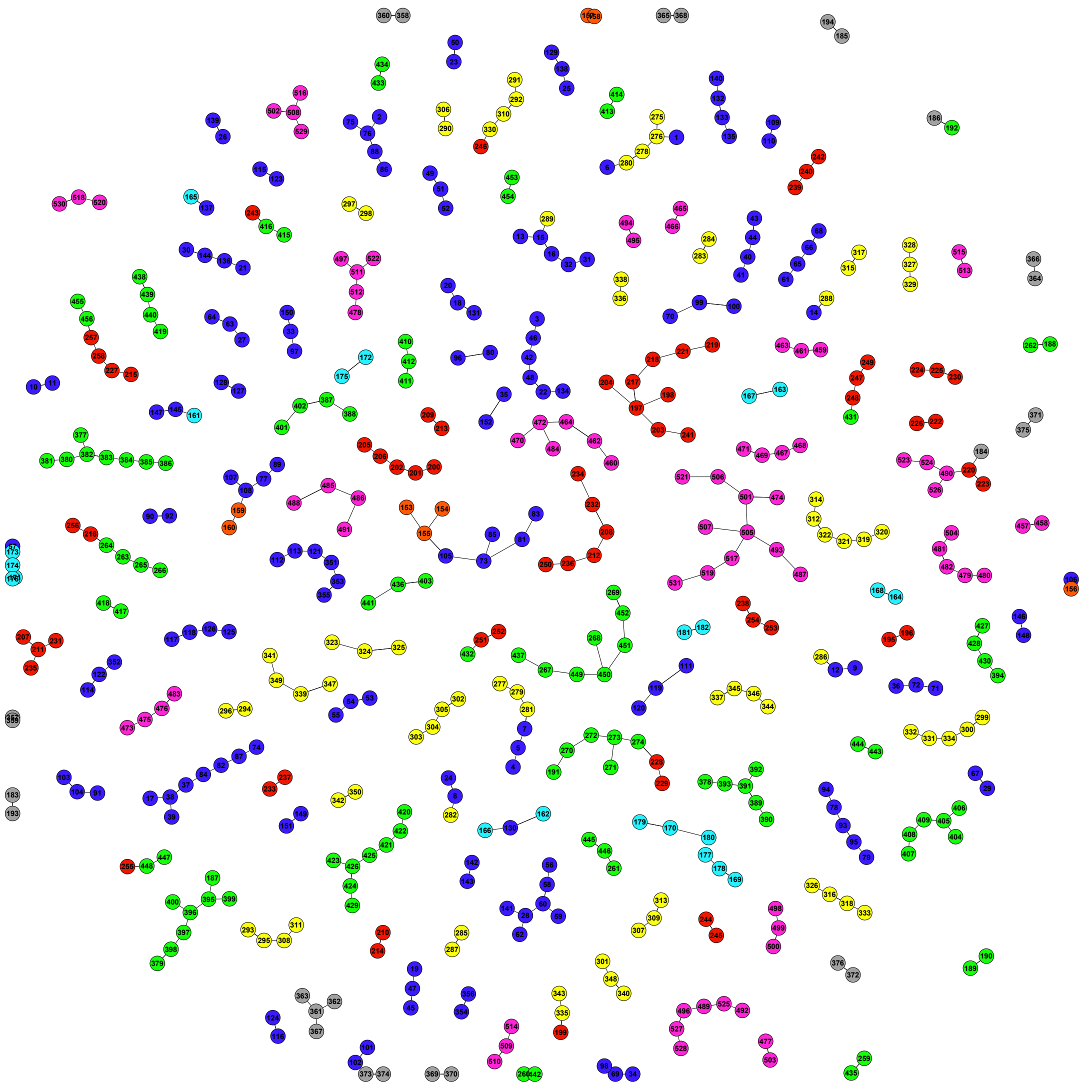

Fig. S10: **Maximum spanning Forest of the real network anatomically parcellized in 531 ROIs.** Arrows indicate that the ROI-source is maximally correlated with the ROI-target.Colors represent anatomical regions according to the grouping of AAL parcellation in 531 ROIs. The anatomical regions are the same of fig. 3(a), the only difference is in the number of ROIs each region is divided into (see Methods for details):● Frontal Lobe; ● Insula; ● Cingulate; ● Temporal Lobe; ● Occipital Lobe; ● Parietal Lobe; ● Deep Grey Matter; ● Cerebellum.

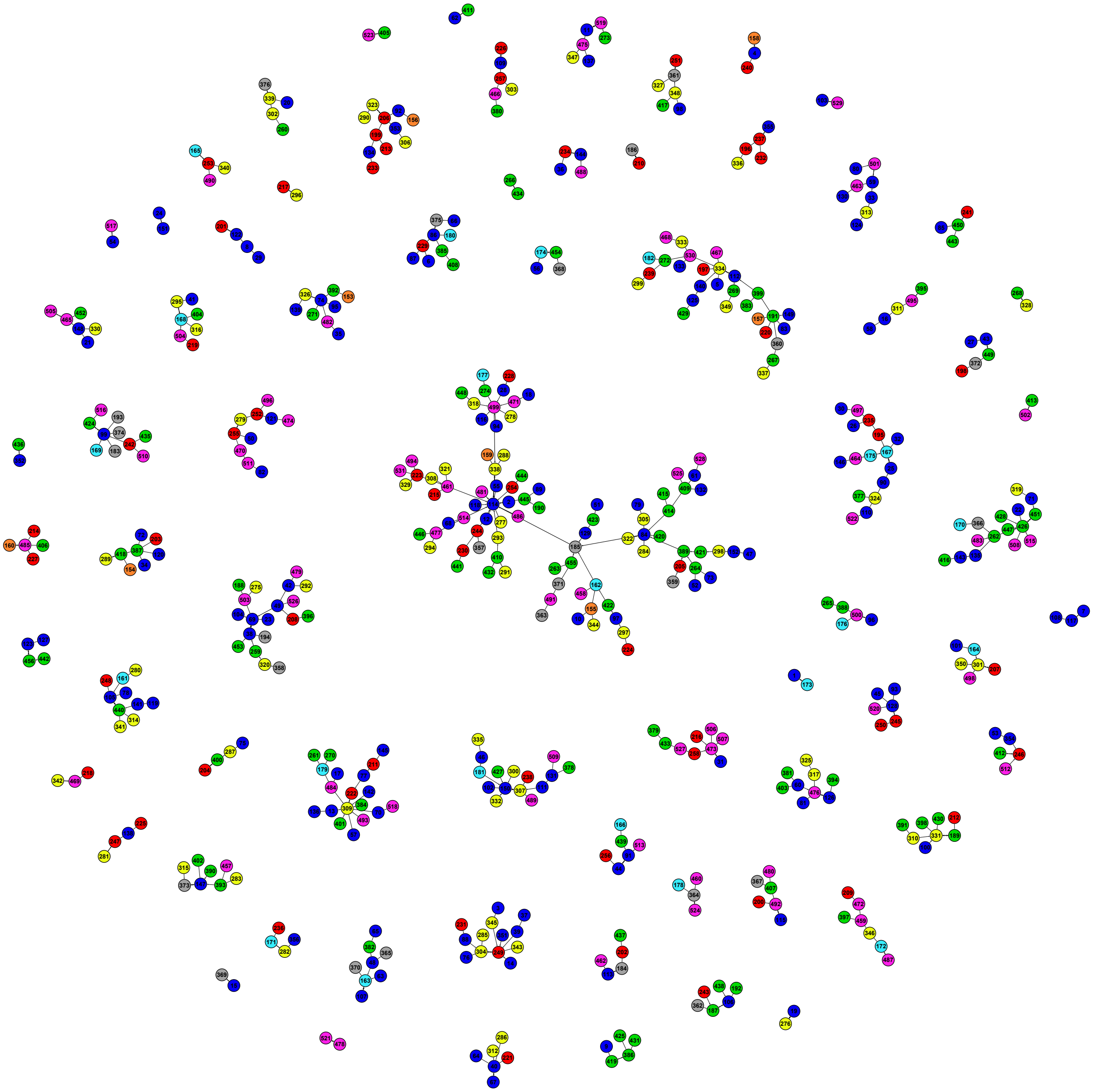

Fig. S11: **Maximum spanning Forest of a randomization of the real network anatomically parcellized in 531 ROIs.** Arrows indicate that the ROI-source is maximally correlated with the ROI-target. Colors represent anatomical regions according to the grouping of AAL parcellation in 531 ROIs. The anatomical regions are the same of fig. 3(a), the only difference is in the number of ROIs each region is divided into (see Methods for details):● Frontal Lobe; ● Insula; ● Cingulate; ● Temporal Lobe; ● Occipital Lobe; ● Parietal Lobe; ● Deep Grey Matter; ● Cerebellum.

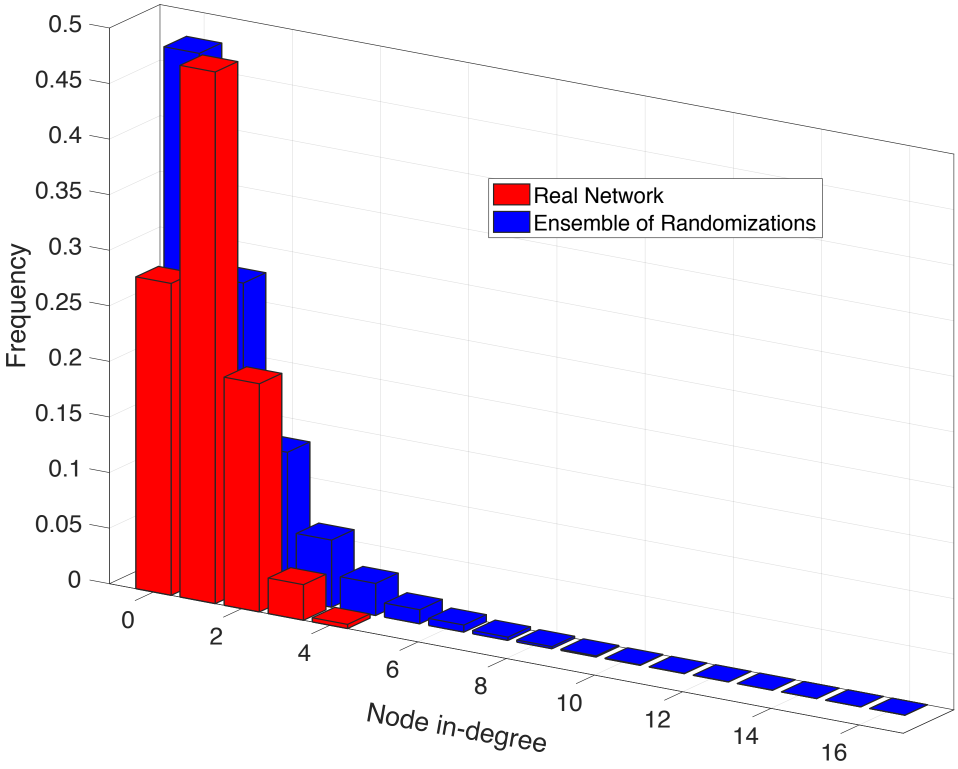


Fig. S12: **Node in-degree distribution of the MSF**. Comparison between the real brain network and the ensemble of its randomizations for the anatomical parcellation of brain in 531 ROIs.

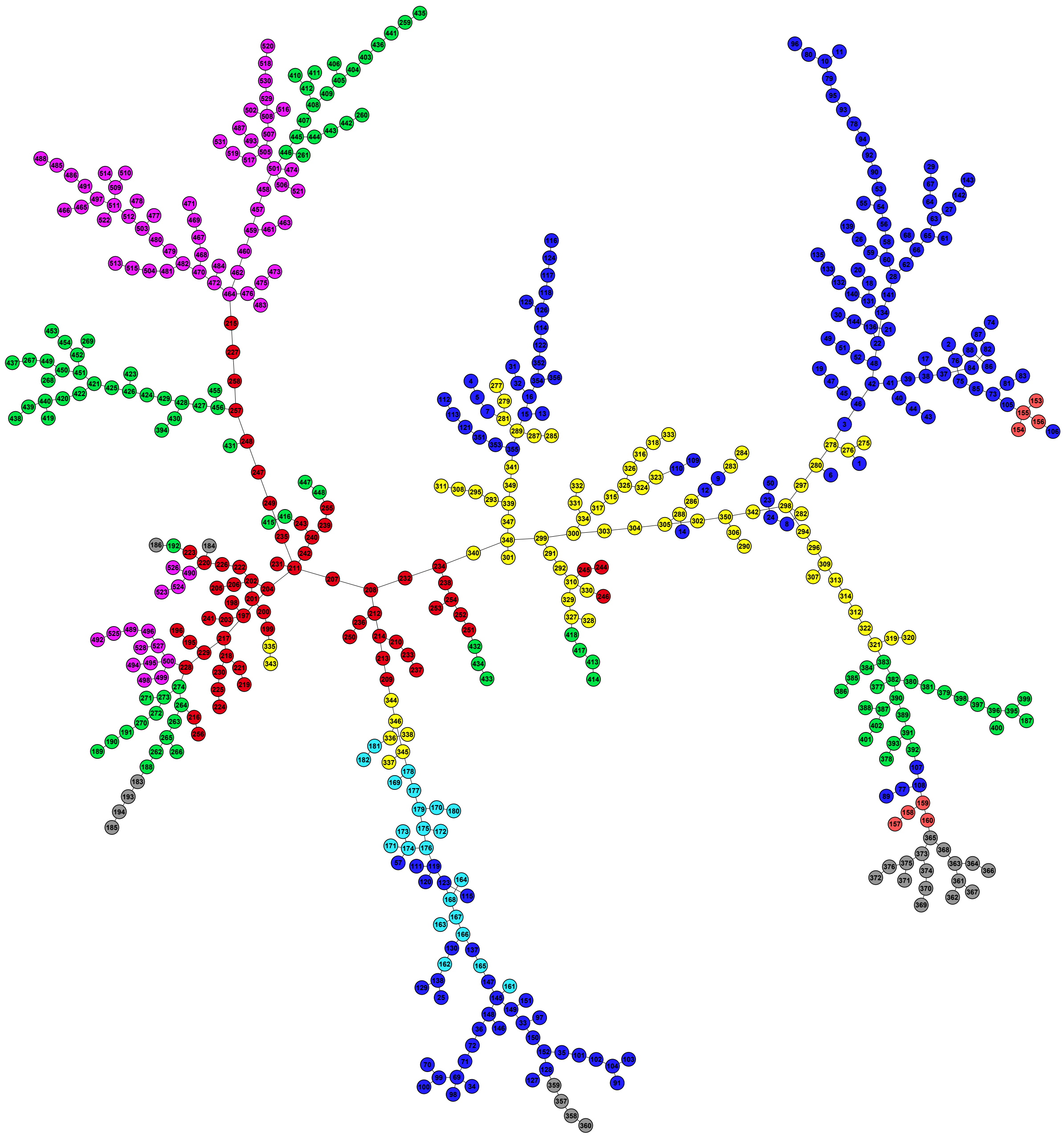

Fig. S13: **Maximum Spanning Tree of the real network anatomically parcellized in 531 ROIs.** Colors represent anatomical regions according to the grouping of AAL parcellation in 531 ROIs. The anatomical regions are the same of fig. 3(a), the only difference is in the number of ROIs each region is divided into (see Methods for details): ● Frontal Lobe; ● Insula; ● Cingulate; ● Temporal Lobe; ● Occipital Lobe; ● Parietal Lobe; ● Deep Grey Matter; ● Cerebellum.

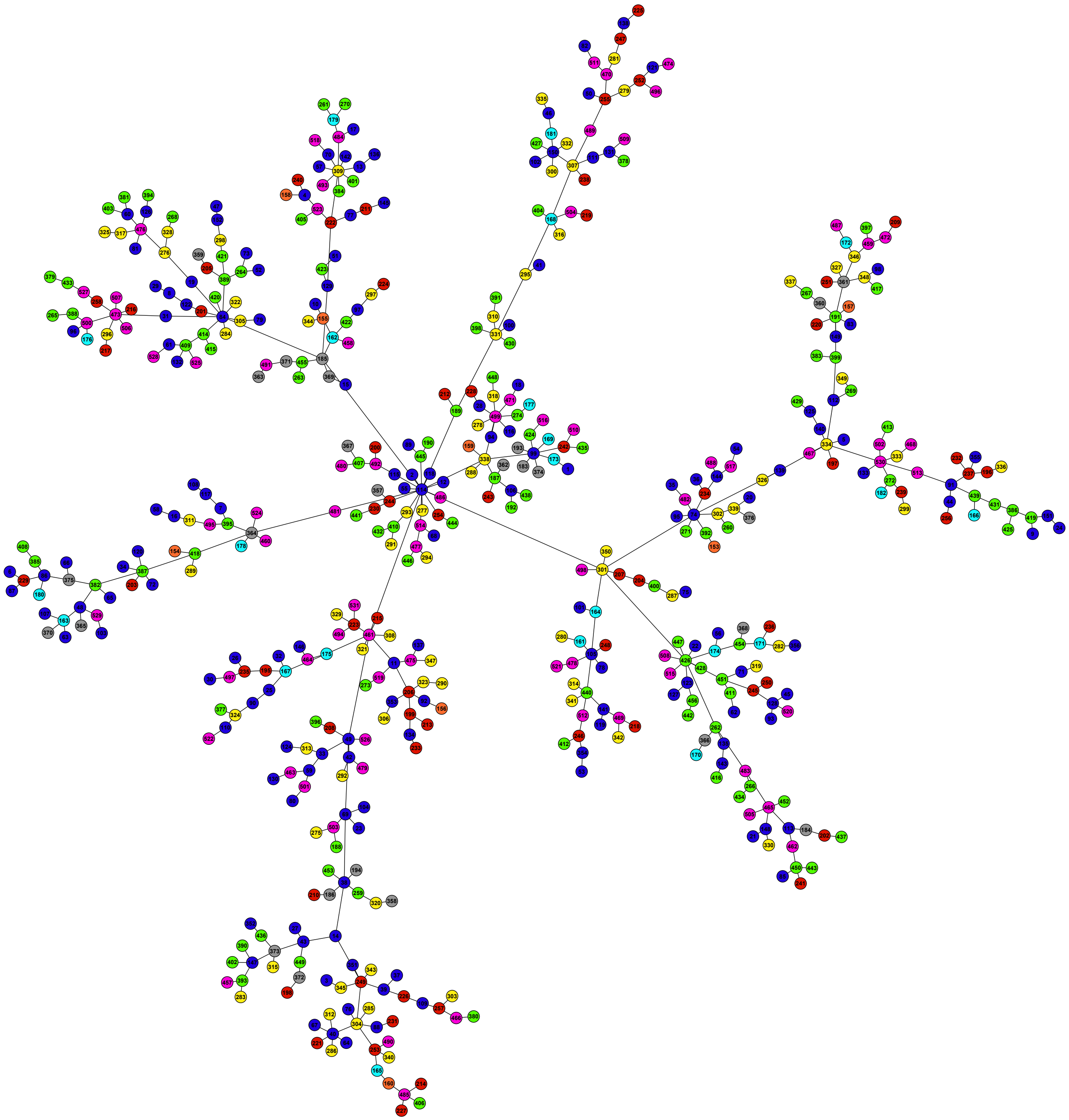

Fig. S14: **Maximum Spanning Tree of a randomization of the real network anatomically parcellized in 531 ROIs.** Colors represent anatomical regions according to the grouping of AAL parcellation in 531 ROIs. The anatomical regions are the same of fig. 3(a), the only difference is in the number of ROIs each region is divided into (see Methods for details): ● Frontal Lobe; ● Insula; ● Cingulate; ● Temporal Lobe; ● Occipital Lobe; ● Parietal Lobe; ● Deep Grey Matter; ● Cerebellum.

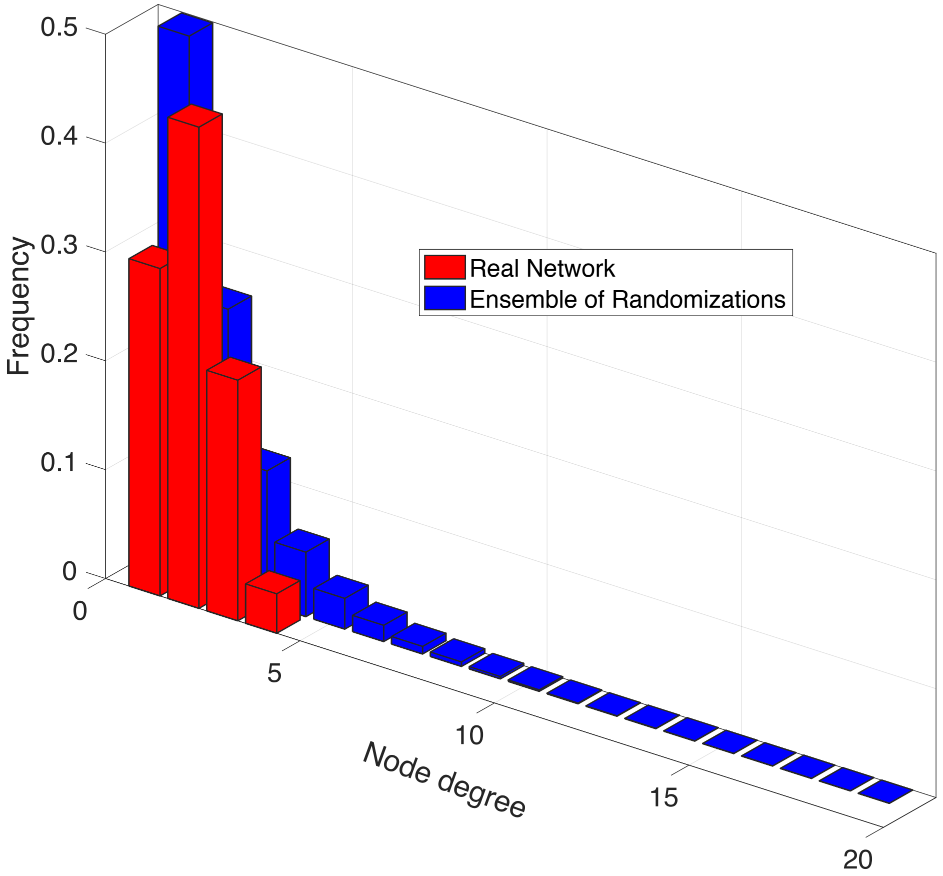


Fig. S15: **Node degree distribution of the MST**. Comparison between the real brain network and the ensemble of its randomizations for the anatomical parcellation of brain in 531 ROIs.
